# Human-in-the-Loop—A Deep Learning Strategy in Combination with a Patient-Specific Gaussian Mixture Model Leads to the Fast Characterization of Volumetric Ground-Glass Opacity and Consolidation in the Computed Tomography Scans of COVID-19 Patients

**DOI:** 10.3390/jcm13175231

**Published:** 2024-09-04

**Authors:** Constanza Vásquez-Venegas, Camilo G. Sotomayor, Baltasar Ramos, Víctor Castañeda, Gonzalo Pereira, Guillermo Cabrera-Vives, Steffen Härtel

**Affiliations:** 1 Department of Computer Science, Faculty of Engineering, University of Concepción, Concepción 4030000, Chile; covasquezv@inf.udec.cl; 2 Laboratory for Scientific Image Analysis SCIAN-Lab, Integrative Biology Program, Institute of Biomedical Sciences, Faculty of Medicine, University of Chile, Santiago 8380453, Chile; camilosotomayor@uchile.cl; 3 Radiology Department, University of Chile Clinical Hospital, University of Chile, Santiago 8380420, Chile; gpr.pereira@gmail.com; 4 School of Medicine, Faculty of Medicine, University of Chile, Santiago 8380453, Chile; baltasarramos@ug.uchile.cl; 5 Center of Medical Informatics and Telemedicine & National Center of Health Information Systems, Faculty of Medicine, University of Chile, Santiago 8380453, Chile; vcastane@gmail.com; 6 Department of Medical Technology, Faculty of Medicine, University of Chile, Santiago 8380453, Chile; 7 Biomedical Neuroscience Institute, Faculty of Medicine, University of Chile, Santiago 8380453, Chile; 8 National Center for Health Information Systems, Santiago 8380453, Chile; 9 Center of Mathematical Modelling, University of Chile, Santiago 8380453, Chile

**Keywords:** COVID-19, Chest CT, Artificial Intelligence, Deep Learning, Human-in-the-Loop, Gaussian Mixture Model

## Abstract

**Background/Objectives:** The accurate quantification of ground-glass opacities (GGOs) and consolidation volumes has prognostic value in COVID-19 patients. Nevertheless, the accurate manual quantification of the corresponding volumes remains a time-consuming task. Deep learning (DL) has demonstrated good performance in the segmentation of normal lung parenchyma and COVID-19 pneumonia. We introduce a Human-in-the-Loop (HITL) strategy for the segmentation of normal lung parenchyma and COVID-19 pneumonia that is both time efficient and quality effective. Furthermore, we propose a Gaussian Mixture Model (GMM) to classify GGO and consolidation based on a probabilistic characterization and case-sensitive thresholds. **Methods:** A total of 65 Computed Tomography (CT) scans from 64 patients, acquired between March 2020 and June 2021, were randomly selected. We pretrained a 3D-UNet with an international dataset and implemented a HITL strategy to refine the local dataset with delineations by teams of medical interns, radiology residents, and radiologists. Following each HITL cycle, 3D-UNet was re-trained until the Dice Similarity Coefficients (DSCs) reached the quality criteria set by radiologists (DSC = 0.95/0.8 for the normal lung parenchyma/COVID-19 pneumonia). For the probabilistic characterization, a Gaussian Mixture Model (GMM) was fitted to the Hounsfield Units (HUs) of voxels from the CT scans of patients with COVID-19 pneumonia on the assumption that two distinct populations were superimposed: one for GGO and one for consolidation. **Results:** Manual delineation of the normal lung parenchyma and COVID-19 pneumonia was performed by seven teams on 65 CT scans from 64 patients (56 ± 16 years old (*μ* ± *σ*), 46 males, 62 with reported symptoms). Automated lung/COVID-19 pneumonia segmentation with a DSC > 0.96/0.81 was achieved after three HITL cycles. The HITL strategy improved the DSC by 0.2 and 0.5 for the normal lung parenchyma and COVID-19 pneumonia segmentation, respectively. The distribution of the patient-specific thresholds derived from the GMM yielded a mean of −528.4 ± 99.5 HU (μ ± σ), which is below most of the reported fixed HU thresholds. **Conclusions:** The HITL strategy allowed for fast and effective annotations, thereby enhancing the quality of segmentation for a local CT dataset. Probabilistic characterization of COVID-19 pneumonia by the GMM enabled patient-specific segmentation of GGO and consolidation. The combination of both approaches is essential to gain confidence in DL approaches in our local environment. The patient-specific probabilistic approach, when combined with the automatic quantification of COVID-19 imaging findings, enhances the understanding of GGO and consolidation during the course of the disease, with the potential to improve the accuracy of clinical predictions.

## 1. Introduction

Computed Tomography (CT) is a fundamental tool for the assessment of disease, as it allows for the visualization of anatomical involvement and the determination of its extent. Furthermore, it is a valuable aid for predicting clinical outcomes [1]. The most prevalent findings in the early stages of the disease are ground-glass opacities (GGOs) and consolidation, with a variable extent that tends to be patchy and bilateral [2,3,4,5]. In GGO, underlying bronchial structures and pulmonary vessels can still be observed through a hazy lung density increment. In contrast, in consolidation these structures are completely obscured [6]. These imaging features manifest differently during disease evolution and are histopathologically associated with degrees of alveolar damage, which in turn influence pulmonary functional involvement and, consequently, the clinical outcome [7]. In the initial stages of the disease, GGO can be observed as a result of the inflammatory process occurring within the parenchyma, which leads to the exudation of fluid into the alveoli and interstitial edema [8,9,10]. As pneumonia progresses, GGOs may transition to consolidation, a process that can be explained by the complete filling of alveolar spaces and interstitial edema. The presence of GGO and/or consolidation has been associated with the severity of a patient’s illness. Pure GGO is observed in patients with a milder illness, while the volume of consolidation progressively increases as the disease progresses. Larger volumes of consolidation are associated with higher odds and unfavorable clinical outcomes in patients [7,11,12].

The quantitative characterization of COVID-19 pneumonia in the lung parenchyma is valuable for predicting the functional progression of the disease and making proactive decisions to reduce the risk of respiratory distress. Manual delineation of regions of interest is a time-consuming and resource-intensive process that relies on the level of expertise of local specialists. To quantify the extent of disease in the parenchyma, automated segmentation methods using deep learning (DL) approaches have been employed [13,14,15,16,17]. The proposal of efficient workflows and interaction between human experts and DL models in Human-in-the-Loop (HITL) methods has been put forth as a means of enhancing the efficiency of the annotation process, with the close monitoring of model quality performance [18,19,20].

DL approaches for the segmentation of COVID-19 pneumonia led to high-quality results [21,22]. However, its success hinges on the availability of extensive training data representative of the local manifestations of the disease. Recommendations exist to identify COVID-19 pneumonia in CT scans by fixed thresholds within the HU, ranging from −368 to −100 HU for non-contrast CT scans [23,24,25,26,27,28,29,30]. Alternative approaches propose the use of clustering methods combined with extensive preprocessing of the CT scans [31,32]. The classification of GGO and consolidation within volumes of COVID-19 pneumonia can be achieved based on a fixed threshold of −300 HU [23,24,25,26,27,28,29,30]. One limitation of this approach is that it might not capture the dynamics of the imaging findings over the course of the disease or variations due to patient characteristics and acquisition protocols.

We tested a HITL strategy using a 3D-UNet [33] initially trained with an international dataset, which underwent iterative retraining cycles with local CT scans. This was carried out on the basis of three hierarchical levels of expertise that improve delineations and generate ground truth labels for the normal lung parenchyma and COVID-19 pneumonia. Furthermore, we propose a voxel-wise probabilistic approach for the segmentation of GGO and consolidation. This is achieved by fitting a GMM [34] to the HU frequency distribution of voxels within COVID-19-infected volumes, and we discuss the use of fixed and patient-specific thresholds for the discrimination of GGO and consolidation. The combination of the HITL strategy with the GMM provides rapid and precise quantitative results, thus enhancing the radiological analysis of CT scans of COVID-19 patients and facilitating the investigation of disease progression based on histopathological criteria.

## 2. Materials and Methods

This research was approved by the Scientific Ethics Committee of the Clinical Hospital of the University of Chile, Certificate No. 35. All CT scans were fully anonymized by the removal of any sensitive information. In accordance with guidelines to enhance reproducibility [35,36], we have provided a report of the TRIPOD checklist in Table S1.

### 2.1. Datasets

Local CT dataset: 65 CT scans (44 non-contrast and 21 contrast-enhanced) acquired with a Siemens SOMATON Definition Edge CT Scanner between March 2020 and June 2021 were randomly selected. Acquisition protocols are shown in Table 1. The first CT scan performed on a total of 64 patients following the suspicion of COVID-19 was used (Table 2). Only one patient underwent both a contrast-enhanced and a non-contrast CT scan.

Seven teams, comprising experts at varying levels of expertise, manually delineated the normal lung parenchyma and COVID-19 pneumonia using the HITL strategy described below. The average percentage of infection of our labeled dataset was 37.5%, ranging from 3.12% to 86.05%. For the HITL strategy, two 3D-Unet models were trained, one for non-contrast and one for contrast-enhanced CT scans; these were trained using 36 non-contrast and 16 contrast-enhanced CT scans, respectively. The remaining 8 non-contrast and 5 contrast-enhanced CT scans were used as a test set, with the objective of evaluating the generalization performance of our model over data not used during the training phase.

International CT dataset (“coronacases” dataset [37]): 10 public non-contrast CT scans were available, with a resolution of 512 × 512 pixels and a slice thickness of 1 mm in 7 samples and 1.5 mm in 3 samples. Each scan was manually delineated to show the normal lung parenchyma and COVID-19 pneumonia. The average percentage of infection was 11.52%, with a range of 0.01% to 59.73%.

**Figure 1:**
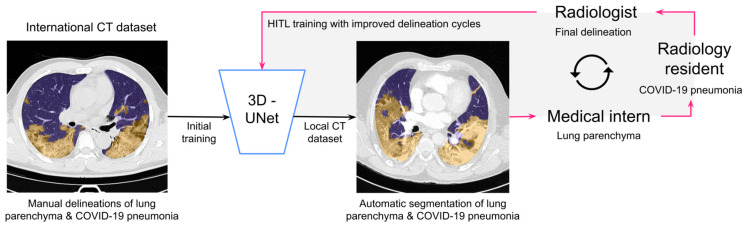
Hierarchical Human-in-the-Loop (HITL) strategy for fast and improved segmentation of normal lung parenchyma and COVID-19 pneumonia through manually corrected delineations by a 3-level hierarchy: (i) basic level for correction of normal lung parenchyma by medical interns; (ii) intermediate level for correction of COVID-19 pneumonia by radiology residents; and (iii) expert level for final corrections or approval by radiologists. HITL cycles permit iterative training of the 3D-UNet with local CT scans, improving the results of the initial training performed using publicly available international CT datasets.

**Figure 2:**
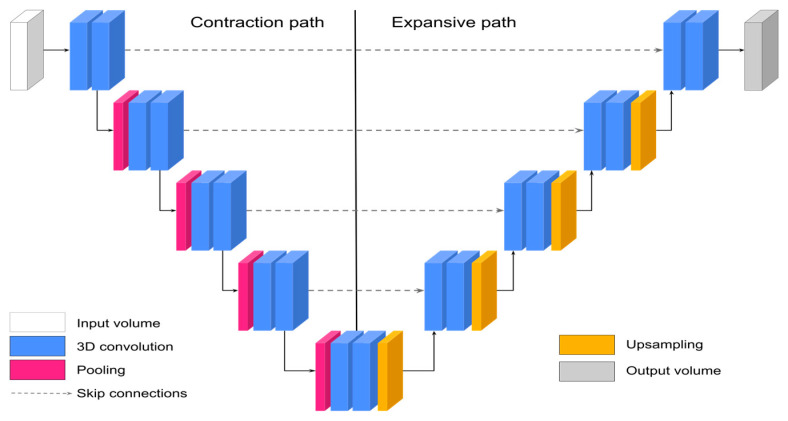
3D-UNet architecture for the segmentation of imaging data. The DL architecture consists of 3D convolutional layers organized in a contraction and an expansive path. The contraction path captures the context and features from the input volume at multiple scales, while the expansive path captures spatial information that was lost across the contraction path. The use of skip connections preserves the content and location of the regions of interest, resulting in the output volume containing a segmentation map.

Local and international CT scans were preprocessed by clipping the voxel intensities between −1250 and 250 HU, normalizing to a grayscale range of [0, 255], and resampling to a target spacing of 1.58 × 1.58 × 2.70 mm based on [15] to ensure homogeneous input conditions for the HITL approach and 3D-UNet training (Figure 1 and Figure 2).

### 2.2. Segmentation of Normal Lung Parenchyma and COVID-19 Pneumonia with 3D-UNet

We performed automatic segmentation using a 3D-UNet [33] (Figure 2), implemented through the TensorFlow-based framework Medical Image Segmentation with Convolutional Neural Networks (MIScnn) [38]. During the training phase, we employed data augmentation techniques [15], including mirroring, scaling [0.85, 1.25], rotation [−15, 15] (degrees), elastic deformation with alpha in the range of [0, 900] and sigma [9, 13], Gaussian noise [0, 0.05], random contrast [0.3, 3], and brightness adjustment [0.5, 2]. To streamline the input data, we randomly extracted 160 × 160 × 80 voxel patches from the original CT scans. The models were trained on an NVIDIA Tesla V100 GPU, provided by Amazon Web Services and the National Laboratory for High-Performance Computing (NLHPC). Training was performed using early stopping, a batch size of 2 patches, a loss function that sums the Tversky index [39] and the categorical cross-entropy, and Adam [40] as the optimization method, as described in [15]. The patient-level cross-validation strategy implemented in [38] was applied to the training set, with 5-fold model validation and selection. The cross-validation strategy partitions data into multiple folds. The model is then trained using *k-1* folds, while the remaining fold is used for validating. This process is repeated until each fold has been used as the validation set. Final evaluations were performed using the test set, which consisted of CT scans that were not seen by the model during the training or validation processes. The quality of the segmentation was evaluated in comparison to the manual delineations in terms of Dice Similarity Coefficients (DSCs) [41] for the normal lung parenchyma and COVID-19 pneumonia.

### 2.3. Human-in-the-Loop (HITL) Strategy for the Fast Optimization and Characterization of Normal Lung Parenchyma and COVID-19 Pneumonia

To ensure the optimal training of DL algorithms, it is essential that critical structures in CT scans are manually delineated by experienced radiologists. As the manual delineation of normal lung parenchyma and COVID-19 pneumonia in CT scans from scratch is time consuming, we propose a hierarchical HITL strategy [27] (Figure 1). Expert teams correct delineation errors from the automatic segmentation results and generate ground truth images. HITL is composed of two phases: the initial prediction of the segmented structure by the automatic segmentation system, followed by the revision and correction of the delineated structure by experts. The initial segmentation results were generated by a 3D-UNet trained on the international dataset [15,37]. Subsequently, the correction process was executed on three levels of expertise: (i) medical interns corrected the normal lung parenchyma; (ii) radiology residents validated the corrections and improved the delineation of COVID-19 pneumonia; and (iii) radiologists performed final corrections. Batches containing the improved delineations were incorporated into the training set for each cycle of the HITL approach. Radiologists established the convergence criteria using a DSC ≥ 0.95/0.8 for the lung parenchyma/COVID-19 pneumonia.

We tested the variation between expert teams in relation to the segmentation of the normal lung parenchyma and COVID-19 pneumonia. Seven expert teams delineated three randomly assigned non-contrast CT scans. For each CT scan, three independent segmentations were obtained. The mean values and corresponding standard variation (*μ* ± *σ*) were derived from a total of 9 DSCs. Volumes of normal lung parenchyma ($V_{L}$) and COVID-19 pneumonia ($V_{I}$) were calculated by the number of voxels and the xyz size of the CT metadata. Percentage of infection (*PI*) was calculated as $PI=\frac{V_{I}}{V_{L}*100}$, and Bland–Altman plots [42] were used to compare the PIs from the automated and manual segmentation.

### 2.4. Gaussian Mixture Model (GMM) to Characterize Ground-Glass Opacity (GGO) and Consolidation

**Figure 3:**
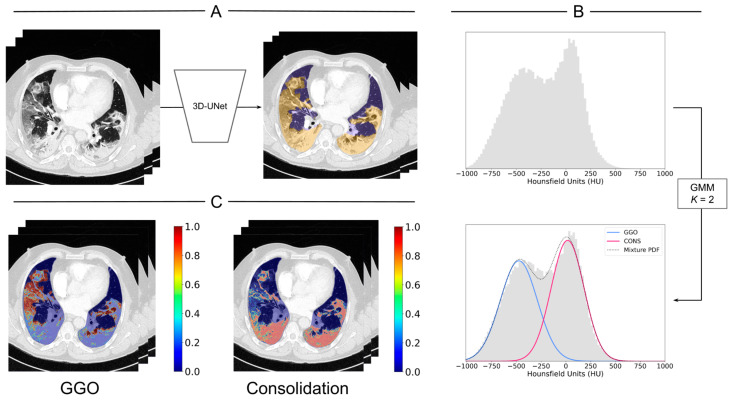
Segmentation of GGO and consolidation in chest CT scans by voxel-wise probabilistic GMM. (**A**) From CT images (**left panel**), normal lung parenchyma (purple and yellow) and COVID-19 (yellow) infection are segmented automatically by a 3D-UNet (**right panel**). (**B**) Area normalized histograms (upper panel) are generated from the voxel intensities (HU) within the COVID-19-infected area, and a GMM (K = 2) is fitted to the data to identify areas with GGO or consolidation (blue and red Gaussian, respectively) together with the mixture probability density function (PDF, gray dotted line). (**C**) A voxel-wise probabilistic characterization is color coded and scaled independently (0–1) for GGO (**left panel**) and consolidation (**right panel**).

We propose a voxel-wise probabilistic GMM for the characterization of GGO and consolidation within areas of COVID-19 pneumonia in volumetric CT scans (Figure 3). Considering the underlying histopathologic processes involved in GGO and consolidation formation, the GMM approximates the distribution of the COVID-19-pneumonia HU intensity values [34]. The GMM works with a univariate mixture of Gaussians distributions, which is composed of *K* normal distributions. Each of these is defined by means ${\left\{\mu_{i}\right\}}_{i=1}^{K}$, variances ${\left\{{\sigma^{2}}_{i}\right\}}_{i=1}^{K},$ and mixing coefficients ${\left\{\pi_{i}\right\}}_{i=1}^{K}$. When the *K* normal distributions are combined, they model the overall distribution of data through a mixture probability density function (PDF). The mixture PDF is $p(x)=\sum_{i=1}^{K}\pi_{i}{Ɲ}_{i}(x|\mu_{i},{\sigma^{2}}_{i})$, where Ɲ is a normal distribution with *x* data points, mean $\mu_{i}$, and variance ${\sigma^{2}}_{i}$. In this work, the Gaussian mixture distribution has *K* = 2 distributions: one for GGO and one for consolidation. The GMM implementation was conducted using the Python library scikit-learn.

**Figure 4:**
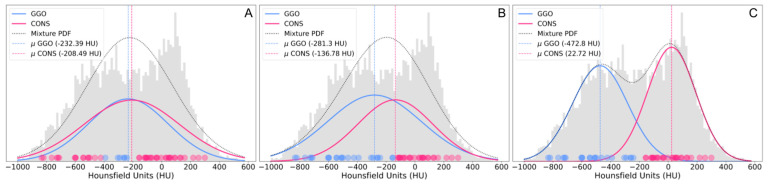
GMM training and optimization for a *K* = 2 population. (**A**) Gaussian distributions for GGO (blue) and consolidation (red) are initialized for the area-normalized Hounsfield histograms (gray), and the probabilities, *p*, for each data point to adhere to GGO or consolidation (low to high opacity for each respective color codifies *p* = 0–1) are computed together with the mixture probability density function (PDF, gray dotted line). (**B**) Mean values, mixing coefficients, and covariances (*μ*, *π*, *σ*^2^) are updated for each iteration, together with the probabilities for each data point. (**C**) Iterations are repeated until convergence of the GMM.

We collected the HU intensity values of the voxels classified as infection by the 3D-UNet and fitted a GMM through the expectation–maximization algorithm independently for each patient’s CT scan. Free parameters were initialized randomly. In the expectation step, the probability of each data point being generated by a specific Gaussian distribution from the mixture of Gaussians is calculated. In the maximization step, the parameters of the distribution were adjusted. The process was repeated until the log-likelihood reached a plateau, indicating convergence (Figure 4). Due to the differences in HU density and texture between GGO and consolidation, we identified each imaging finding in the distribution using the means defined by the model. The lower mean value was associated with GGO [23,24,25,26,27,28,29,30]. Using voxel probabilities, color maps for GGO and consolidation were generated (Figure 3C). Probability distributions and patient-specific thresholds for GGO and consolidation were computed independently for each CT scan in a patient-specific manner, based on the HU intensity value of voxels with a 0.5 probability for either of the two classes: GGO or consolidation. In the absence of ground truth labels for GGO and consolidation, the method was assessed in comparison with fixed thresholds that have been previously reported [23,24,25,26,27,28,29,30].

## 3. Results

A total of 65 CT scans of 64 patients diagnosed with COVID-19 pneumonia were analyzed. The patients presented to the Emergency Department with at least one symptom and were 56 ± 16 years old (*μ* ± *σ*); 72% were male, 17% had a history of diabetes mellitus, and 41% had hypertension. The majority of patients (88%) were admitted to the hospital, with 42% of these patients requiring admission to the Intensive Care Unit. The median overall in-hospital length of stay was 21 days (IQR, 0–84), and this was 14 days (IQR, 0–73) for patients admitted to the Intensive Care Unit. In total, 11 patients died during in-hospital admission (Table 2).

The implemented HITL strategy (Figure 1) met the quality criteria established by local radiologists in only three HITL cycles. The teams corrected 21 CT scans for the first HITL cycle, 14 CT scans for the second, and 12 for the third and final cycle. Segmentation results improved with each iteration. Manual delineations of normal lung parenchyma and COVID-19 pneumonia of the expert teams were used as ground truths for calculating the quality of segmentation with the DSC (Table 3). The performance of the HITL approach in terms of the DSC for non-contrast and contrast-enhanced CT scans yielded values that were superior to the coefficients defined by radiologists (DSC ≥ 0.95/0.8 for normal lung parenchyma/COVID-19 pneumonia). The DSCs increased with each cycle, with higher DSCs obtained for the normal lung parenchyma in comparison to COVID-19 pneumonia for non-contrast and contrast-enhanced CT scans. For comparison purposes, we determined the DSC among seven expert teams who performed manual segmentation of the normal lung parenchyma and COVID-19 pneumonia in non-contrast CT scans from scratch in randomly assigned non-contrast CT scans. The DSC yielded 0.98 ± 0.01 for the normal lung parenchyma and 0.86 ± 0.03 for COVID-19 pneumonia (*μ* ± *σ*), which was comparable to or within the quality of the automated segmentation achieved after the third HITL cycle.

**Figure 5:**
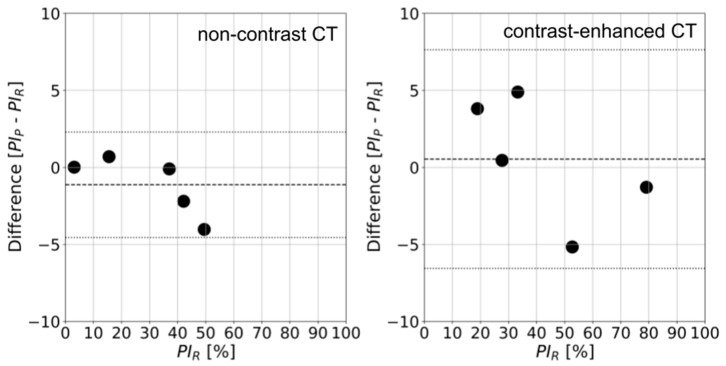
Differences in the percentage of infection calculated for the HITL approach (*PI_P_*) and the standard of reference percentage (*PI_R_*) for COVID-19 pneumonia for non-contrast (**left panel**) and contrast-enhanced CT scans (**right panel**) after three HITL cycles. Mean values and 95% confidence intervals are plotted as dashed and dotted lines, respectively.

Bland–Altman plots show differences in the percentage of infection, calculated from the results obtained after three HITL cycles and the delineations of expert teams for COVID-19 pneumonia (Figure 5). The error margin for non-contrast and contrast-enhanced CT test images was within 5%. For non-contrast and contrast-enhanced CT, the mean differences and 95% confidence intervals were −1.12% [−4.55%, 2.30%] and 0.54% [−6.56%, 7.64%], respectively.

**Figure 6:**
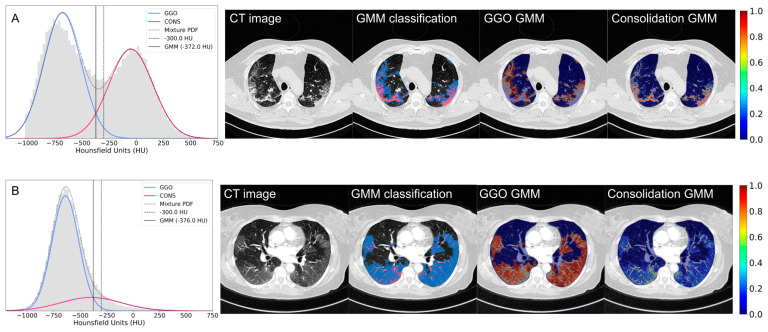
Patient-specific characterization of GGO and consolidation using a GMM. (**A**) Non-contrast CT scan with balanced voxel populations for GGO and consolidation. (**B**) Contrast-enhanced CT scan where GGO voxels outweigh consolidation. For A and B, area-normalized histograms (gray) in HU with Gaussian distributions for GGO (blue) and consolidation (red) are shown with mixture PDF (dotted line), fixed threshold at −300 HU (dashed line), and CT patient-specific GMM thresholds (black line). For A and B from left to right, we show original CT images, color-coded results obtained by GMM classification at variable HU threshold (GGO in blue and consolidation in red), and GGO and consolidation GMM with corresponding color-coded probability scale *p* = 0–1.

Following the successful training and validation of the HITL approach for the segmentation of COVID-19 pneumonia in non-contrast and contrast-enhanced CT scans, we proceeded to address the challenge of classification and quantification of GGO and consolidation within areas of COVID-19 pneumonia. Figure 3, Figure 4 and Figure 6 show the results for the segmentation of GGO and consolidation in chest CT scans using a voxel-wise probabilistic GMM approach. Figure 4 shows the application of a two-component GMM (*K* = 2), representing GGO and consolidation, along the mixture of both Gaussian distributions as a mixture PDF. A GMM is fitted to the HU of voxels within the segmented COVID-19 pneumonia region. The initial mean values, mixing coefficients, and covariances (Figure 4A) are updated with each iteration (Figure 4B) until the final convergence of the GMM (Figure 4C), which allows for the calculation of the probabilistic characterization and voxel-wise color coding for GGO and consolidation (Figure 3).

**Figure 7:**
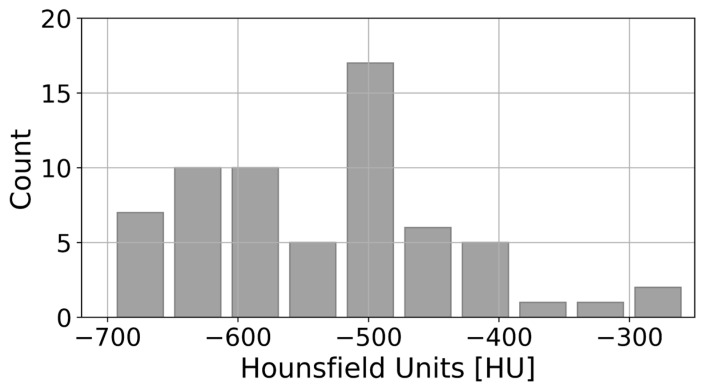
Histogram of patient-specific thresholds based on GMM for 64 CT scans from the local dataset, including non-contrast and contrast-enhanced CT scans.

The final results of the GMM for GGO and consolidation in contrast-enhanced and non-contrast-enhanced CT scans, together with the patient-specific thresholds derived from the GMM, are presented in Figure 6. Figure 6A presents a representative example of a nearly balanced COVID-19 pneumonia in terms of the number of voxels representing GGO or consolidation areas (55 vs. 45%). In Figure 6B, voxels associated with GGO strongly outweigh consolidation (89 vs. 11%). In both cases, the patient-specific thresholds are less than −300 HU (−372 HU for A and −376 for B). The distribution of the patient-specific thresholds for 65 CT scans yielded −528.4 ± 99.5 HU (*μ ± σ*). Patient-specific thresholds (Figure 7) exhibited a range from a minimum value of −697 HU to a maximum value of −256 HU, contingent on the stage of pneumonia progression of the individual patients.

While the HU threshold leads to a binary classification of each voxel (see GMM classification images in Figure 6A,B), the patient-specific GMM provides two probability estimates for each voxel identified as COVID-19 pneumonia (see voxel-wise color coding for GGO GMM and consolidation GMM in Figure 6A,B). The voxel-wise color coding, in conjunction with the patient-specific threshold, facilitates the identification of regions associated with the transition of the histopathologic processes of the disease. The voxel-wise color coding for the consolidation GMM in Figure 6B shows that lung blood vessels are predominantly classified as consolidation, with a lower probability of being identified as GGO (comparing red and blue coded voxels in the respective CT images).

## 4. Discussion

The HITL approach, trained with the international dataset and re-trained with local CT scans, demonstrated an improvement in the DSC for automatic segmentation by ~0.2 for the normal lung parenchyma and ~0.5 for COVID-19 pneumonia for non-contrast and contrast-enhanced CT scans. Improving the quality of segmentation for a local CT dataset holds clinical promise with the potential to improve the accuracy of clinical predictions, which remains to be further studied. The approach accelerated the annotation process, minimizing the time that expert teams dedicated to manual delineation. The final DSCs of the automatic segmentation were found to be similar to those determined among seven expert teams (0.98 ± 0.01 for the normal lung parenchyma and 0.86 ± 0.03 for COVID-19 pneumonia (*μ* ± *σ*), which proves that HITL cycles are not only time efficient, but also accurate. The formation of teams comprising three levels of expertise contributed to the advancement of knowledge and skills, which is of particular importance in the context of emerging diseases and the need for rapid adaptation to protocols within a value chain.

A recent review of HITL machine learning [18] categorizes the approaches into three main groups: active learning (system in control), interactive machine learning (interaction between users and system), and machine teaching (domain experts have control over the learning process). Our approach combines interactive machine learning with a teaching methodology. Domain experts, including interns, residents, and radiologists, utilize the outcomes and oversee the quality of the visualization and DSC. Residents and radiologists participated in the design, development, and training phases of the system, actively contributing regarding usability, design, and color representation. Our results also support the reported key benefit [19] of including end users in DL supported systems in terms of the best data selection, interaction with GMM outputs, consideration of the daily clinical practice, and future prospective. Finally, our findings are in line with those in [20] in terms of shortcutting time-intensive annotations in CT scans for the spleen, liver, kidneys, stomach, gallbladder, pancreas, aorta, or IVC. The use of HITL cycles proved effective in reducing bias among expert teams, detecting automated delineation errors, and enabling teams to identify and rectify the most salient mistakes.

The 3D-Unet HITL training cycles yielded a DSC on non-contrast CT scans for the normal lung parenchyma and COVID-19 pneumonia that was in close agreement with the results of seven expert teams. The third HITL cycle fulfilled the expert quality criteria and exceeded our expectations. The DSCs for the normal lung parenchyma in non-contrast and contrast-enhanced CT scans show comparable performance. The DSC for COVID-19 pneumonia provides a higher DSC on contrast-enhanced CT scans, due to the medium that enhances the pattern in the CT images. Non-contrast CT scans for the normal lung parenchyma segmentation showed higher DSC values (0.97 ± 0.02) with respect to the best DSC value (0.86 ± 0.1) reported with a similar approach [14]. COVID-19 pneumonia in non-contrast CT (0.82 ± 0.12) was lower than the highest DSC (0.92 ± 0.1) reported in [27], but higher than the values reported in [14,15] (0.67 ± 0.22, 0.76). To the best of our knowledge, DSCs have not yet been reported for COVID-19 pneumonia in contrast-enhanced CT scans.

The automatic calculation of PI enriches the radiological report by adding quantitative information about the extent of COVID-19 pneumonia signs. Currently, quantitative information in terms of volumes or PI is not routinely included in the radiological reports, with only subjective extent or visual estimation being employed. Our findings suggest that mean values of *PI_P_*–*PI_R_* exhibit a 1% deviation from zero, both in non-contrast and contrast-enhanced CT scans, representing a low deviation. According to the participating radiologists, the mean error and confidence interval suggest that the proposed method could be used in clinical practice after clinical validation.

In addition to the results of the HITL cycles, in terms of DSC and PI for the automatic segmentation of normal lung parenchyma and COVID-19 pneumonia, the GMM offers a probabilistic characterization of GGO and consolidation in the CT scans of COVID-19 patients. Within the voxels identified with COVID-19 pneumonia, HU histograms show patterns that suggest two underlying Gaussian distributions (Figure 3B, Figure 4 and Figure 6). HU arbitrarily scales physical attenuation coefficients of distilled water to 0, and air to −1000 at standard pressure and temperature, representing a relative quantitative unit for radio density, since the density of tissue is proportional to the attenuation of X-ray beams [43]. To our knowledge, there is no universally accepted standard for HU thresholds for GGO or consolidation. Indeed, a review of the literature reveals a wide range of reported values [23,24,25,26,27,28,29,30,43]. In [23], they defined a HU lower than −300 for GGO, −300 to 50 for sub-solid tissue, and greater than 50 for solid tissue. Similarly, the authors in [24,26] used HU thresholds for GGO at −703/−368, and consolidation at −100/5. In the study on HU thresholds by [25], the highest diagnostic efficacy and effectiveness for GGO was reported at a HU lower than—300. Issues of concerns for a standard HU are raised in [43], such as X-ray beam energy variations, CT parameters, or CT artifacts like beam-hardening, which might affect tissue absorption and hence HU.

Patient-specific HU thresholds based on a GMM allows for characterization of the infection (Figure 6A,B). This approach facilitates the identification of regions that are more likely to manifest as GGO or consolidation, as well as the detection of transitions between GGO and consolidation. A dynamic threshold can be calculated for voxel-wise binary classification into GGO, or consolidation based on probabilities, which allows quantification in response to local conditions. Our patient-specific thresholds based on the GMM yielded −528.4 ± 99.5 HU (*μ* ± *σ*), on average ∼75% below −300 HU (Figure 7). Clinical correlation of the imaging findings identified with the proposed method is a potential avenue for further study.

COVID-19 can manifest with a wide spectrum of radiological findings, which vary according to the stage of the disease. We analyzed a patient cohort of the first stage of the pandemic, with a high number of patients with symptoms, lung parenchyma disease, and clinical burden, particularly with high in-hospital and Intensive Care Unit admission, and death events. Therefore, lung parenchyma disease evaluated on CT scans may represent changes secondary to acute COVID-19 pneumonia with a higher likelihood compared to the post-vaccine era, wherein chronic lung parenchyma changes may be found on imaging. Whether this may decrease the prognostic value of automatic lung parenchyma quantification in the acute setting warrants further evaluation.

The transition of GGO to consolidation is gradual, and there is no exact visual threshold that can be used to define the borders in CT scans. Delineation may vary according to the slice thickness, the acquisition protocol, the patient-specific morphometric composition, the degree of pulmonary involvement, the viewing window levels, and the particular CT scanner vendor/model.

The HITL-GMM approach is robust to accommodate these many variables in the quantification of lung parenchyma disease. However, there are still some limitations that require attention. Firstly, it is acknowledged that the reliance on retrospective data in this study may potentially introduce a degree of selection bias, given that the dataset was collected over a limited period of time and may not fully represent the population of patients confirmed as having COVID-19 at the included institution. Secondly, the HITL approach was designed for implementation in a specific health institution; the results may not be generalizable to other institutions or to patients with different demographic or clinical characteristics. Thirdly, as previously stated, the current study focused on the acute phase of COVID-19, with data collected during the early stages of the pandemic. It is currently unclear as to whether our findings can be applied to the post-vaccine era. Finally, the segmentations made by physicians may be biased due to inter- and intra-observer variability, as well as inherent technical factors such as the presence of artifacts.

It would be beneficial for future research to validate our approach with larger, multi-centric datasets, including longitudinal studies to assess the clinical significance of the HITL-GMM method over time. Additionally, we can explore the use of radiomics combined with machine learning models, as its potential capacity to predict the risk of occurrence of clinical outcomes has been demonstrated [44,45,46]. The efficiency of HITL cycles could be improved by ranking and prioritizing samples through attention maps calculated in terms of inconsistency, uncertainty, and overlap to enhance regions that radiologists should focus on for correction [20]. Integration into clinical workflows and PACS, along with the development of a user-friendly interface, will be essential for the broad adoption and clinical validation of this quantitative platform.

## Figures and Tables

**Table 1:** Acquisition protocols for 44 non-contrast and 21 contrast-enhanced CT scans.

Parameters	Non-Contrast CT	Contrast-Enhanced CT
Voltage [kV]	120	80
Rotation time [s]	0.5	0.33
Slice thickness [mm]	1.5	1.5
Collimation [mm]	0.6	0.6
Image resolution	512 × 512	512 × 512
Slides reconstruction	1.5 × 1	1.5 × 1
Algorithm	70f	I70f

**Table 2:** Demographics and clinical variables of patients with COVID-19 pneumonia.

Variable	*n* (%)
Male	46 (71.9)
No symptoms *	0 (0)
Fever *	29 (45.3)
Cough *	47 (73.4)
Expectoration *	13 (20.3)
Dyspnea *	54 (84.4)
Diabetes mellitus	11 (17.2)
Hypertension	26 (40.6)
Chronic obstructive pulmonary disease	0 (0)
Cardiovascular disease	5 (7.8)
Hospitalization	56 (87.5)
Intensive care unit admission	36 (56.3)
Death	11 (17.2)
Hospitalization days **	20.5 [0, 84]
Intensive care unit days **	13.6 [0, 73]

* Clinical symptoms with 2 missing values. All others have 1 missing value. ** Hospitalization and intensive care unit days are reported as mean [min, max].

**Table 3:** Quality of 3D-UNet segmentation at initial training and third HITL cycle over test set, and agreement between expert teams over three independent CT scans.

	DSC (*μ* ± *σ*) 3D-UNet (Initial Training)		DSC (*μ* ± *σ*) 3D-UNet (3 HITL Cycles)		DSC (*μ* ± *σ*) Expert Teams
	Non-Contrast CT	Contrast-Enhanced CT	Non-Contrast CT	Contrast-Enhanced CT	Non-Contrast CT
Normal lung parenchyma	0.77 ± 0.15	0.84 ± 0.03	0.97 ± 0.02	0.97 ± 0.03	0.98 ± 0.01
COVID-19 pneumonia	0.31 ± 0.19	0.41 ± 0.14	0.82 ± 0.12	0.90 ± 0.11	0.86 ± 0.03

## Data Availability

Available upon request to the authors.

## Supplementary Materials

The following supporting information can be downloaded at: https://www.mdpi.com/article/10.3390/jcm13175231/s1, Table S1: TRIPOD Checklist—Prediction Model Development with page indication for each item.
